# Associations of common infections with frailty and mortality in two UK cohort studies

**DOI:** 10.1093/gerona/glag046

**Published:** 2026-02-15

**Authors:** Demelza Smeeth, Charlotte Warren-Gash, Rebecca E Green, Julia Butt, Tim Waterboer, Alun D Hughes, Nishi Chaturvedi, Dylan M Williams

**Affiliations:** Unit for Lifelong Health and Ageing at UCL, University College London, London, United Kingdom; Faculty of Epidemiology and Population Health, London School of Hygiene and Tropical Medicine, London, United Kingdom; Unit for Lifelong Health and Ageing at UCL, University College London, London, United Kingdom; Division of Infections and Cancer Epidemiology, German Cancer Research Center, Heidelberg, Germany; Division of Infections and Cancer Epidemiology, German Cancer Research Center, Heidelberg, Germany; Unit for Lifelong Health and Ageing at UCL, University College London, London, United Kingdom; Unit for Lifelong Health and Ageing at UCL, University College London, London, United Kingdom; Unit for Lifelong Health and Ageing at UCL, University College London, London, United Kingdom; Division of Psychiatry, University College London, London, United Kingdom

**Keywords:** Frailty, Longevity, Infection, Epidemiology

## Abstract

**Background:**

Some common infections are associated with poorer age-related health outcomes; however, findings are limited to a small number of pathogens and frequently inconclusive. This study aimed to expand the range of pathogens investigated in relation to frailty and mortality in older age.

**Methods:**

We investigated relationships between seropositivity for 18 viruses, bacteria and protozoa with concurrent frailty and prospective mortality in middle-aged and older adults within two UK population‐based cohorts: UK Biobank (*N *= 9427; aged 40-70 years) and Medical Research Council National Survey of Health and Development (*N *= 1791; aged 60-65 years). Multiplex serological assays were used to identify seropositivity for each pathogen and frailty was assessed using a frailty index measuring the accumulation of age-related health deficits. Mortality was determined from linked administrative records.

**Results:**

Adjusting for sex, age, income and education, previous infection with *Toxoplasma gondii* ((*β* = 0.77%; 95% CI, 0.42-1.11) and *Helicobacter pylori* (0.63%; 95% CI, 0.28-0.97) were associated with higher frailty equivalent to 3.8 or 3.1 years of aging, as was inflammation-weighted pathogen burden (0.41%/SD, 95% CI, 0.25-0.57; 0.42%/SD, 95% CI, 0.26-0.58). Previous infection with *Chlamydia trachomatis*, human herpes simplex virus 1 and cytomegalovirus were associated with increased frailty after adjustment for sex and age, although relationships were confounded by socioeconomic circumstances. No common infections were robustly associated with mortality.

**Conclusions:**

Our results indicate that infection with *H. pylori* and *T. gondii*, and the combined burden of infection may detrimentally impact ageing health. These pathogens may warrant targeting beyond current clinical measures to mitigate the development of frailty.

## Introduction

The global increase in ageing populations has led to an interest in understanding the development and prevention of frailty and other age-related outcomes.^1^ Frailty reflects an individual’s reduced physiological reserve and increased vulnerability to a wide range of adverse health outcomes, including mortality^2^ and can be assessed through the construction of a frailty index (FI).^3^ This construct uses cumulative counts of age-related health deficits across multiple biological systems, encompassing physical, psychological, biological and social functioning and are associated with a number of negative outcomes including mortality and hospitalization.^4^^,^^5^ Understanding the factors that contribute to frailty and mortality is essential for developing strategies to promote healthy ageing and reduce healthcare burdens.

Many common pathogens have been linked to health beyond the initial acute infection phase. Some, including those from the *Herpesviridae* family, can establish lifelong latency following primary infection.^6^ Others, such as *Helicobacter pylori,* may persist chronically and have significant long-term health consequences.^7^ These infections can adversely affect health directly, through tissue damage and cellular disruption, or indirectly, by promoting systemic inflammation, immune dysregulation, or autoimmunity. Despite the high global prevalence of these persistent infections, the extent to which individual pathogens influence frailty and mortality risk remains poorly understood. Existing studies have explored a narrow range of pathogens and have yielded mixed findings. For example, cytomegalovirus (CMV) seropositivity has been associated with increased frailty and mortality in some populations, but not in others, with differences potentially influenced by sample size, cohort age, health status, and geographic or socioeconomic factors.^8–13^ Limited numbers of studies have begun to expand the range of pathogens explored, but require replication.^14–16^

Cumulative pathogen burden, a composite measure of exposure to multiple pathogens, has also been linked to frailty and mortality, but research remains sparse.^16^^,^^17^ Moreover, the optimal approach to assessing cumulative pathogen exposure requires evaluation. Simple count scores employed to date might have biased any associations of pathogen burden with health towards the null because pathogens with varying pathogenicity are given equal weighting. A more appropriate scoring system would ideally account for differences in pathogenicity of each agent being studied, potentially weighting via their respective inflammatory responses or other underlying shared pathogenic pathways.

In the present study we build on existing research and expand the range of pathogens investigated in relation to frailty and mortality in two large population-based studies in older age. We first examined cross-sectional associations of serostatus for 17 individual pathogens with concurrent frailty, measured using FIs. Second, we explored whether serostatus for these pathogens were also associated with incident all-cause mortality risk. Finally, we explored whether the cumulative burden of these pathogens was associated with frailty or mortality.

## Methods

### Cohorts

The UK Biobank (UKB) is large prospective multicenter cohort study with 502,631 participants, aged 40-69 years, enrolled at 22 assessment sites in England, Scotland, and Wales between 2006 and 2010.^18^ Baseline assessments captured a wide range of data including demographic, health outcome and biological data. This study used the random subset (*n* = 9427; 4151 male, 5276 female) whose baseline blood samples were previously assayed for serology.^19^ This random subset is representative of the wider UKB cohort.

The Medical Research Council 1946 National Survey of Health and Development (NSHD) is a birth cohort study which has followed an initially nationally representative sample of 5362 individuals since their births in England, Scotland, and Wales in one week in March 1946.^20^ This research used data on the subset of the cohort (*n* = 1791; 885 male, 906 female) who participated in the 2006-2010 follow-up at age 60-65 years. This follow-up was chosen due to the availability of recent and current health data for FI generation, collected blood samples for serology assays and similarity of age to UKB.

All cohort participants provided written informed consent. Ethical approval for UKB from the National Health Service North-West Research Ethics Committee (11/NW/0382) and for NSHD was obtained from the National Research Ethics Service Committee London (14/LO/1173).

### Multiplex serology and pathogen serostatus

Serum immunoglobulin G antibody levels against a selection of antigens from several pathogens (herpesviruses, polyomaviruses, papillomaviruses, bacteria and a protozoan) were measured using a validated fluorescence bead‐based multiplex serology platform developed at the German Cancer Research Center in Heidelberg.^19^ Antibody responses were quantified as median fluorescence intensity units, and between one and six antigens were quantified per pathogen (Table S2). In UKB, 21 pathogens were assayed at the baseline assessment. An adaptation of the same multiplex panel assayed 18 pathogens in samples from the NSHD 2006-2010 assessment. Only the 17 pathogens with relevant serology data available in both cohorts were included in this study.

Our primary exposures of interest were serostatus, categorized as binary variables, indicating current and/or past infection for each pathogen (seropositivity); these have been previously derived for both UKB and NSHD (see “the Methods section”).^19^^,^^21^ To measure cumulative exposure to multiple pathogens, we derived a pathogen burden index (PBI) representing the number of positive serostatus values across the 17 pathogens. Indices of pathogen burden have been frequently used to explore the link between infection load and ageing.^16^^,^^17^ We also created novel inflammation-weighted PBIs by weighting each pathogen by its association with either circulating white blood cell count (WBC) or ln-transformed C-reactive protein (CRP) measured from the same blood samples. While inflammation-weighted PBIs are novel to this study, PBIs weighted by other factors have been used successfully to explore disease-weighted pathogen load in the context of stroke and cognitive functioning.^22^^,^^23^ Regression coefficients for each pathogen were obtained from linear regression models adjusted for age and sex and applied as weights to the corresponding pathogen values and summed to derive the final WBC- or CRP-weighted PBIs for each individual (Table S3). PBIs were normalized and the *z*-scores used in all analyses.

### Frailty indices

Frailty indices (FIs) have been previously generated for the two cohorts detailed here using data from the baseline UKB questionnaire and self-report items from the age 60-64 NSHD data collection.^5^^,^^24^ For this analysis, these were modified to create new FIs with alignment across the cohorts and following recent guidance on FI creation.^3^ Items were retained from the existing FIs if they were common to both cohorts, were similarly defined and adhered to the guidelines outlined by Theou et al.^3^ (no more than 10% missing values, and prevalences greater than 1% and less than 80% in both cohorts). Due to differences in data collected in the two cohorts, some items were adapted to improve consistency. Available data for the two cohorts were screened for further potential FI items. New items had to measure an age- or mortality-related health deficit and adhere to the same rules outlined above. All items were scaled from 0 to 1, where 0 represents no health deficit and 1 represents the maximum deficit. In total, this resulted in 41 items present in both cohorts, which covered several health domains including pain, cardiometabolic health, infirmity, mental health and respiratory health. The final items were screened to ensure there were no collinear items using Pearson correlation (*r* < 0.95). Further information on FI creation is outlined in the Supplementary Methods. FI items were scored for each individual, divided by the maximum score (41) and multiplied by 100 to give a percentage FI with 0% representing no frailty and 100% representing maximum frailty.

### Mortality data

Participants in UKB and NSHD provided consent for their data to be linked to the NHS registers for mortality registrations. Mortality data is available up to August 2024 for NSHD and up to November 2021 for UKB.

### Additional covariates

Cohort participants reported sex and age at assessment. Ethnicity was reported only in UKB as White (reference level), Asian, Black, Mixed, or Other. Participants in NSHD were exclusively White. In UKB gross household tax before tax was categorized into <£18 000 (ref.), £18 000-30 999, £31 000-51 999, £52 000-100 000, or >£100 000. Net household income was reported in NSHD and was categorized into <£14 999 (ref.), £15 000-£29 999, £30 000-£39 999, £40 000-£79 999, or >£80 000. Highest level of education was selected from a list of options in UKB: none (ref.), vocational, sub GCE, O-Level, A-Level, degree, or higher, or other professional qualifications. Highest level of education in NSHD was partially collapsed in NSHD to yield similar categories to UKB: none (ref.), vocational, sub GCE, O-Level, A-Level, or degree or higher.

### Statistical analyses

All analyses were conducted in R (v. 4.4) in RStudio.

Missing covariates and FI items were imputed using multiple imputation by chained equations using the ‘mice’ package (v. 3.17) in R, using 20 iterations and 20 chains.^25^ Numerical and binary variables were imputed using predictive mean matching (pmm), ordered factor variables were imputed using the proportional odds model (polr) and unordered factor variables were imputed using polytomous logistic regression (polyreg). Auxiliary variables included body mass index (BMI), smoking status (current/past/never), childhood social class (NSHD only), age at leaving education, employment status, and Townsend deprivation index quintile for their residence at time of interview. Passive imputation was applied to the BMI FI item, PBI, and FI. Convergence was visually assessed through trace-plots, and imputed values were compared with observed data using various diagnostic plots to ensure validity.

Due to differences across studies, analyses were run independently in each individual cohort prior to meta‐analysis. Analyses in NSHD were conducted using the available sampling weight to account for the socially stratified design.^26^ All analyses were conducted individually for each pathogen and PBI. All primary descriptive and inferential analyses were conducted across 20 imputed datasets, which were combined using Rubin’s rules. Sensitivity analyses were performed on complete-case datasets to evaluate robustness of findings. Due to the multiple tests performed, findings from meta‐analyses (per outcome) were corrected for false discovery rate (FDR), using the Benjamini–Hochberg procedure with an alpha of 0.05. However, given that multiple testing correction increases the likelihood of false negative results, suggestive findings (at unadjusted *p *< .05) in fully adjusted models are also described.

### Frailty analyses

We used multivariable linear regression models to examine the cross-sectional association of each individual pathogen serostatus or PBI with frailty. All models included the individual pathogen serostatus or PBI as the exposure and FI percentage as the outcome. Minimally adjusted models controlled for participant sex and age at assessment, as well as ethnicity in UKB. Fully adjusted models additionally controlled for income and educational attainment as potential confounders. Outputs are regression betas which represent the adjusted difference in FI percentage in seropositive vs seronegative groups (for individual serostatuses) or per standard deviation increase in PBI with 95% confidence intervals.

The average yearly increase in frailty was calculated to contextualize the results of regression models. This was calculated using data from UKB only due to the limited and skewed age range covered by NSHD. Average yearly increases in frailty were estimated from linear models which modelled the relationship between age at interview and frailty percentage controlling for sex and ethnicity.

### All-cause mortality analyses

We constructed Cox proportional hazard regression models with the “survival” package (v. 3.17) to examine the association of each individual pathogen and PBI with risk of all-cause mortality.^27^ The proportional hazards assumption was tested for each model and proportional hazards were assumed where *p* > .05. In these models, age at baseline assessment was used as the starting time, and age at censoring or death was used as the time-to-event variable. Models were adjusted as for frailty analyses. Outputs are hazard ratios which represent the adjusted relative hazards of death during follow-up in seropositive versus seronegative groups (for individual serostatuses) or per standard deviation increase in PBI.

### Meta-analysis

Two separate random-effects meta-analyses were performed using the rma function from the “metafor” package (v.4.8):^28^ one synthesizing linear regression coefficients and one synthesizing hazard ratios derived from Cox proportional hazards models. Due to differences in demographics and follow-up periods across the studies, we fitted a random-effects model using the restricted maximum likelihood (REML) estimator, which provides approximately unbiased estimates of between-study variance compared to other methods such as maximum likelihood. REML estimation enables study weights to vary according to both within-study precision and the estimated between-study variance, preventing any single study from dominating the pooled effect when heterogeneity is present. Regression betas and standard errors were provided for FI analyses. Log-hazards and their accompanying standard errors were provided for survival analyses. Substantial heterogeneity between cohorts was defined as *I*^2^ > 50% and/or *Q p* value < .05. We also inspected the direction of individual study estimates. Meta-analyzed results for survival analyses were exponentiated to provide hazard ratios for reporting.

## Results

### Cohort descriptions

Full descriptions of the cohorts can be found in Table 1. Both cohorts were balanced for sex (UKB 56.0% female; NSHD 49.4% female) and comprised adults over the age of 40 years (UKB mean age at baseline: 56.5 years, SD 8.2; NSHD mean age at 60-64 years follow-up: 63.2 years, SD 1.1).

**Table 1:** Cohort descriptions for UK Biobank and NSHD.

	UK Biobank		NSHD	
	(*n* = 9427)		(*n* = 1791)	
**Age, mean years (SD)**		56.5 (8.2)		63.2 (1.1)
**Female sex, *N* (%)**		5276 (56.0)		885 (49.4)
**Ethnicity, *N* (%)**	White	8921 (94.6)	White^a^	–
	Asian	233 (2.5)		
	Black	142 (1.5)		
	Mixed	53 (0.6)		
	Other	78 (0.8)		
**Income^b^, *N* (%)**	<£18 000	2315 (24.6)	<£15000	431 (24.1)
	£18 000-30 999	2453 (26.0)	£15 000-£29 999	758 (42.3)
	£31 000-51 999	2411 (25.6)	£30 000-£39 999	260 (14.5)
	£52 000-100 000	1777 (18.9)	£40 000-£79 999	270 (15.1)
	>£100 000	471 (5.0)	>£80 000	72 (4.0)
**Education (%)**	None	1654 (17.5)	None	511 (28.5)
	Vocational	634 (6.7)	Vocational	53 (3.0)
	Sub GCE	530 (5.6)	Sub GCE	87 (4.9)
	O-Level	2036 (21.6)	O-Level	374 (20.9)
	A-Level	1053 (11.2)	A-Level	542 (30.3)
	Degree, other professional qualifications, or higher	4090 (43.4)	Degree or higher	224 (12.5)
**Frailty index %, median (IQR)**		13.4 (8.5, 19.9)		15.8 (10.8, 22.3)
**Died during follow-up, *N* (%)**		715 (7.6)		280 (15.6)

Descriptive statistics pooled from imputed datasets. Percentages may not add up to 100% due to rounding.

Abbreviations: GCE, General Certificate of Education; NSHD, National Survey of Health & Development.

a Data not collected but based on migration patterns over 20th century it can be assumed that NSHD was predominantly White British.

b Gross household income in UK Biobank, net household income in NSHD. Equivalent net income brackets for UK Biobank: <£14 344, £14 344-£23 314, £23 314-£36 990, £36 990-£65 310, and >£65 310.

Seroprevalence of the pathogens studied were generally consistent across the two cohorts, although prevalence for a small number of pathogens was lower in NSHD (Table 2). Most infections were relatively common except for herpes simplex virus 2 (HSV2), Kaposi’s sarcoma-associated herpesvirus, and the papillomaviruses (seroprevalences ≤16.2%). Accordingly, total pathogen burden was relatively high with most participants testing positive for at least seven pathogens (UKB mean = 9.0; NSHD mean = 7.4).

**Table 2:** Seroprevalence for the 17 pathogens and pathogen burden indices.

		UK biobank	NSHD
		(*n* = 9427)	(*n* = 1971)
**Herpesviruses, %**	HSV1	69.9	67.3
	HSV2	16.2	7.3
	VZV	92.4	79.4
	EBV	94.7	92.9
	CMV	58.3	54.2
	HHV6A	77.4	42.7
	HHV6B	79.2	53.9
	HHV7	94.7	72.8
	KSHV	8.1	0.3
**Papillomaviruses, %**	HPV-16	4.4	2.7
	HPV-18	2.7	2.4
**Polyomaviruses, %**	BK virus	95.3	91.4
	JC virus	57.5	51.6
	MC virus	66.5	59.7
**Bacteria/protozoa, %**	*T. gondii*	28.0	24.4
	*H. pylori*	31.7	17.9
	*C. trachomatis*	21.5	17.6
**Pathogen burden indices, mean (SD)**	Unweighted PBI	8.98 (2.07)	7.39 (1.98)
	WBC-weighted PBI	0.82 (0.24)	0.28 (0.34)
	CRP-weighted PBI	0.36 (0.15)	−0.16 (0.10)

Values pooled from imputed datasets.

Abbreviations: *C. trachomatis*, *Chlamydia trachomatis*; CMV, cytomegalovirus; CRP, C-reactive protein; EBV, Epstein–Barr virus; *H. pylori*, *Helicobacter pylori*; HHV, human herpesvirus; HPV, human papillomavirus; HSV, herpes simplex virus; JC, John Cunningham virus; KSHV, Kaposi’s sarcoma–associated herpesviruses; MCV, Merkel cell virus; NSHD, National Survey of Health & Development; PBI, pathogen burden index; *T. gondii*, *Toxoplasma gondii*; WBC, white blood cells.

FIs were comparable across the cohorts although slightly higher in NSHD as expected given the older mean age of the participants (UKB: median FI: 13.4%, IQR: 8.5, 19.9; NSHD: median: 15.8%, IQR: 10.8, 22.3; Table 1; Figure S1). Accordingly, many of the individual FI items had higher mean scores in NSHD indicating greater frailty according to that health deficit (Table S5). The FI values and distributions were similar to those observed in other longitudinal population-based studies from the UK.^29–31^ FIs were positively correlated with age in UKB (*rs *= 0.19, 95% CI, 0.17-0.21) and associated with increased risk of all-cause mortality in both cohorts (UKB: HR = 1.05 per percentage higher FI, 95% CI, 1.04-1.06; NSHD: HR = 1.04, 95% CI, 1.03-1.05) indicating the validity of the measure.^3^ The FI was not correlated with age in NSHD due to the limited age range covered.

The mean follow-up time was 12.5 years (SD = 0.13) in UKB and 14.3 years (SD = 1.6) in NSHD. Within this study period, 715 (7.6%) UKB participants and 280 (15.6%) NSHD participants had died.

### Pathogen seropositivity and frailty

**Figure 1:**
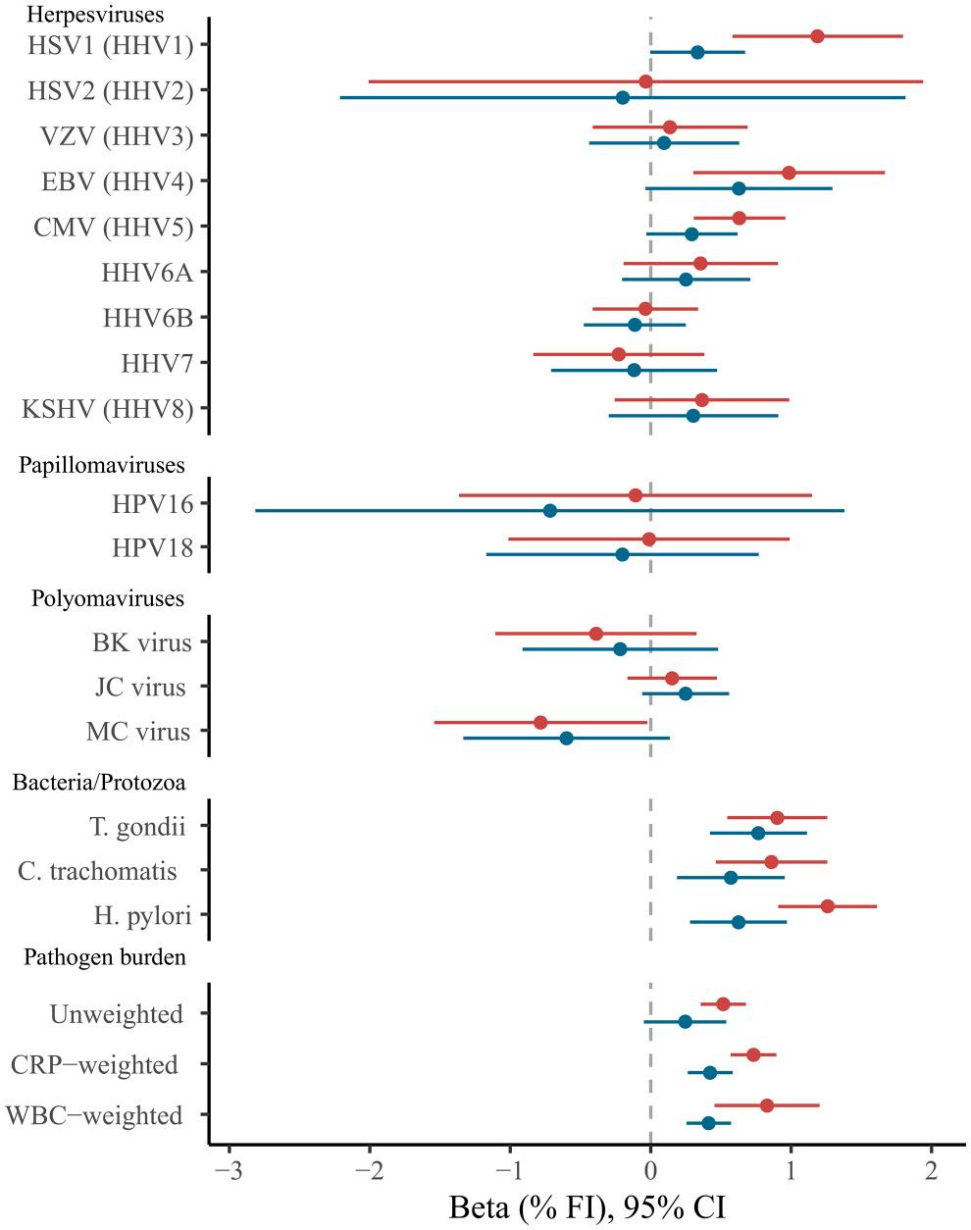
Forest Plot indicating meta‐analyzed associations of pathogen serostatus and pathogen burden scores with frailty. Beta coefficients represent the mean change in frailty index (%) between the exposed and reference groups for individual pathogens, and for each SD increase in PBI. Positive coefficients indicate increased frailty. Minimally adjusted models (upper bars) controlled for sex, age, and ethnicity in UKB. Fully adjusted models (lower bars) additionally controlled for income and educational attainment. Pathogen families are indicated. CMV, cytomegalovirus; CRP, C-reactive protein; EBV, Epstein–Barr virus; HHV, human herpesvirus; HPV, human papillomavirus; HSV, herpes simplex virus; JC, John Cunningham virus; KSHV, Kaposi’s sarcoma–associated herpesviruses; MCV, Merkel cell virus; WBC, White blood cell.

Meta-analyzed results for associations of individual pathogen serostatus with frailty are depicted in Figure 1 and full results are in Tables S6 and S7. Seropositivity for several common infections was associated with higher frailty when compared to seronegative individuals, including *Toxoplasma gondii* (*β* = 0.90%; 95% CI, 0.55-1.27), *H. pylori* (*β* = 1.26%; 95% CI, 0.90-1.61), *Chlamydia trachomatis* (*β* = 0.86%; 95% CI, 0.45-1.61), human herpes simplex virus 1 (HSV1) (*β* = 1.19%; 95% CI, 0.58-1.80), and CMV (*β* = 0.63%; 95% CI, 0.31-0.96). These point estimates were equivalent to the differences in frailty accrued over 3.1-6.2 years of ageing in UKB. In addition, seropositivity for Epstein–Barr virus and Merkel Cell virus were associated with increased and decreased frailty respectively, but these did not survive controlling for multiple testing and the results for Merkel Cell virus showed high between-study heterogeneity (*I*^2^ = 62.5). We note that the mean coefficients for EBV’s association with frailty were comparable to that of *H. pylori* and *T. gondii* but were imprecisely estimated due to there being few seronegative individuals in the samples.

Many of these relationships were attenuated with adjustments for socioeconomic factors. After controlling for income and education, only *Toxoplasma gondii* (*β* = 0.77%; 95% CI, 0.42-1.11) and *H. pylori* (*β* = 0.63%; 95% CI, 0.28-0.97) were significantly associated with frailty. These coefficients are equivalent to an increase of frailty seen over 3.8 and 3.1 years of ageing respectively. Seropositivity for *Chlamydia trachomatis* was nominally associated with frailty, but this did not survive controls for multiple testing (*β* = 0.57%; 95% CI, 0.19-0.95).

In minimally adjusted models, all PBIs were associated with higher frailty, with the inflammation-weighted PBI associations slightly more pronounced (PBI_unwt_: *β* = 0.52%/SD, 95% CI, 0.36-0.68; PBI_WBC_: *β* = 0.83%/SD, 95% CI, 0.45-1.20; PBI_CRP_: *β* = 0.73%/SD, 95% CI, 0.57-0.89). The associations of all three PBIs with frailty attenuated with adjustment for socioeconomic covariates, though statistically robust evidence remained for the associations of inflammation-weighted PBIs (PBI_unwt_: *β* = 0.25/SD, 95% CI, −0.05, 0.55; PBI_WBC_: *β* = 0.41%/SD, 95% CI, 0.25-0.57; PBI_CRP_: *β* = 0.42%/SD, 95% CI, 0.26-0.58).

Associations between serostatus variables and frailty were generally consistent in direction between the two cohorts, although effect sizes were often smaller in NSHD. There was no evidence of heterogeneity where pooled results were statistically significant, but heterogeneity was evident for other pathogens (ie, where *I^2^* > 50% or *Q *< 0.05, Tables S6 and S7). Most notably, HSV2 seropositivity was associated with higher frailty in UKB, but lower frailty in NSHD. For pooled findings, UKB tended to contribute a much greater proportion to the weighting of overall estimates, likely reflecting its larger sample size. Associations estimated from imputed datasets were also generally consistent with complete-case analyses although confidence intervals were substantially narrower, particularly for very rare or common pathogens (Table S10).

### Pathogen seropositivity and all-cause mortality

**Figure 2:**
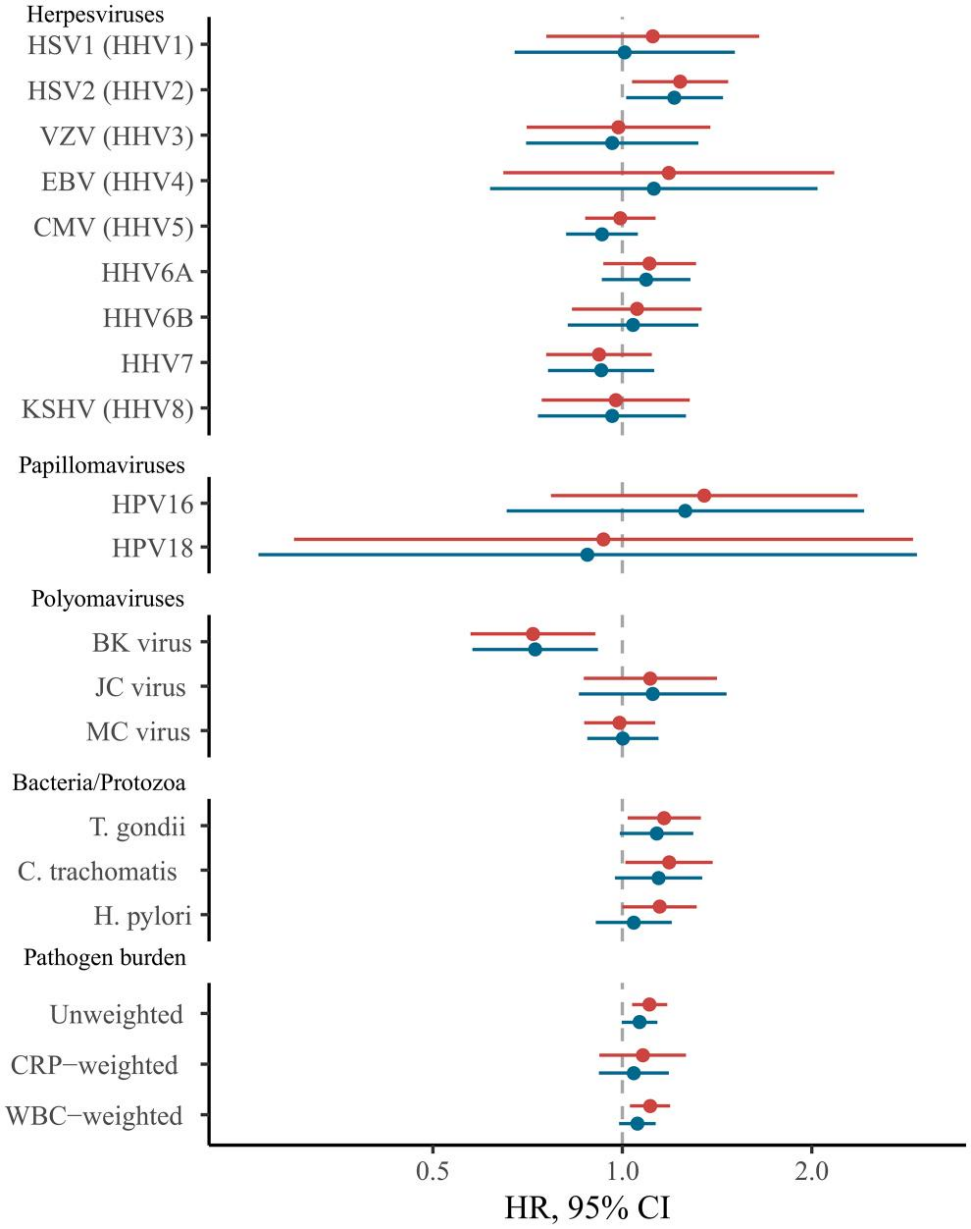
Forest plot indicating meta‐analyzed associations of pathogen serostatus and pathogen burden scores with risk of mortality. Hazard ratios (HR) represent the mean change in mortality risk between the exposed and reference groups for individual pathogens, and for each SD increase in PBI. Positive estimates indicate increased risk of mortality. Minimally adjusted models (upper bars) controlled for sex, and ethnicity in UKB. Fully adjusted models (lower bars) additionally controlled for income and educational attainment. Pathogen families are indicated. Mortality events total 715 (7.6%) in UKB and 280 in (15.6%) NSHD. CMV, cytomegalovirus; CRP, C-reactive protein; EBV, Epstein–Barr virus; HHV, human herpesvirus; HPV, human papillomavirus; HSV, herpes simplex virus; JC, John Cunningham virus; KSHV, Kaposi’s sarcoma–associated herpesviruses; MCV, Merkel cell virus; WBC, White blood cell.

Compared to frailty, there were fewer clear associations of common pathogens with risk of all-cause mortality over a mean follow-up of 13 years (Figure 2; Tables S8 and S9). In minimally adjusted models, only the unweighted PBI was significantly associated with 11% higher risk of mortality after controlling for multiple testing (HR = 1.11, 95% CI, 1.04-1.18). The WBC-weighted PBI (HR = 1.11, 95% CI, 1.03-1.19), HSV2 (HR = 1.24, 95% CI, 1.04-1.47), *T. gondii* (HR = 1.17, 95% CI, 1.02-1.3), and *C. trachomatis* (HR = 1.19, 95% CI, 1.01-1.39) were associated with higher mortality risk prior to multiple testing correction while BK virus was associated with a 28% lower risk of mortality (HR = 0.72, 95% CI, 0.57-0.91).

Following adjustment for socioeconomic factors and correction for multiple testing, no serology measures were significantly associated with mortality. However, HSV2 seropositivity was nominally associated with an elevated higher risk of mortality (HR = 1.21, 95% CI, 1.01-1.44), while BK virus seropositivity was nominally associated with a lower risk of mortality (HR = 0.73, 95% CI, 0.58-0.91). Associations from imputed datasets were generally consistent with complete-case analyses (Tables S12 and S13).

## Discussion

In this cross‐cohort study using two British population‐based samples of middle-aged to older adults, we found that exposure to several common infections was associated with increased frailty. Of these the clearest evidence of individual pathogen associations with frailty was observed for two species of bacteria (*H. pylori and C. trachomatis)* and a protozoan (*T. gondii*). *C. trachomatis* and *H. pylori* are responsible for some of the most frequent chronic infections in humans, infecting the mucosal cells in genital tracts and ocular systems, and the stomach respectively.^32^^,^^33^ Despite their prevalence, symptomatic infections are scarce, but these pathogens are associated with elevated peripheral inflammation which has been hypothesized to lead to more systemic impacts in addition to known clinical consequences, such as pelvic inflammatory disease and peptic ulcer disease. To our knowledge, this is the first investigation of *H. pylori* and *C. trachomatis* exposure in relation to frailty, although *H. pylori* infection has previously been linked to multi-system morbidity and mortality.^34^

Infections with the protozoan *T. gondii* are also common (seroprevalences >20% in both current cohorts) often establishing latent and asymptomatic infections in multiple tissues. While more commonly associated with neuropsychological outcomes,^35^ latent *T. gondii* infection has been associated with elevated allostatic load, a factor involved in the development of frailty.^36^ This is consistent with a previous small case-control study, which found a positive association between seroreactivity and frailty among *T. gondii* seropositive individuals.^15^

Our findings provide initial evidence for the potential long-term population health impact of common pathogens such as *H. pylori, C. trachomatis*, and *T. gondii*. To date, vaccines for these pathogens have not been approved for use in humans, therefore prevention would typically aim to minimize transmission through public health interventions. *H. pylori* is predominantly transmitted via the oral-oral or facal-oral routes, therefore improving sanitation and reducing household crowding is recommended.^32^  *C. trachomatis* is transmitted almost exclusively through sexual contact, therefore prevention typically focusses on sexual health education and routine screening.^37^  *T. gondii* has a zoonotic life cycle, with felines as the primary reservoir, therefore preventative interventions include improving food safety and promoting safe handling of cat litter.^38^ While eradication therapies can successfully clear *H. pylori* and *C. trachomatis*, their use is generally limited to those that seek clinical treatment or are screened for infection.^37^^,^^39^ Promoting early detection and eradication therapy may also help reduce the long-term effects of infections without overt symptoms. In contrast, there is currently no approved therapy that can eradicate the tissue cysts responsible for chronic *T. gondii* infection limiting the options for intervention post-infection.

Previous research on the association between pathogen serostatus and frailty has tended to focus predominantly on herpesviruses, and particularly CMV given its relationship to the ageing immune system.^40^ Such studies suggest that herpesviruses may be associated with increased frailty, but associations are inconsistent.^16^^,^^40–42^ In this study, while CMV, HSV1, and EBV were associated with higher frailty in minimally adjusted models, this relationship attenuated with adjustment for socioeconomic covariates (particularly in NSHD), potentially explaining the inconsistent findings across different populations or settings. However, some caution is needed regarding the interpretation of models adjusted for socioeconomic factors: whilst these factors could plausibly confound associations, it is also possible that infections may mediate some of the effects of education and other socioeconomic conditions on these health outcomes. We note that *C. trachomatis and H. pylori* remained associated with frailty regardless of an individuals’ socioeconomic circumstances, strengthening our conclusion that these species may directly impact frailty in infected individuals.

Despite several associations of pathogen serostatus with frailty, there was limited evidence for associations with all-cause mortality over the study period. Results for HSV2, *T. gondii and C. trachomatis* were all at least suggestive of higher mortality risk among seropositive individuals, while BK virus was suggestive of reduced mortality risk among seropositive individuals. The plausibility of the relationships of *T. gondii* and *C. trachomatis* with mortality risk is increased by their clear parallel associations with frailty and limited existing research.^43^ A number of studies have found reduced survival rates in seropositive individuals for some of the pathogens studied, particularly for CMV, *H. pylori* and HSV2,^9^^,^^10^^,^^13^ although not consistently.^13^^,^^34^^,^^44^ It has been previously noted that a positive association between CMV serostatus and mortality often arises in studies which have larger sample sizes or a greater number of mortality events than was available in this work.^14^ Unexpectedly, BK virus was associated with reduced mortality risk among seropositive individuals. It is not clear how this association might arise. Assuming it is not a chance finding and given infection is common in childhood, it is possible that it is linked to the long lag time between initial infection and the period over which mortality was studied. Alternatively, it may be a consequence of some biasing mechanism linked to non-exposure to BK virus, or survivorship bias if BK virus increased mortality prior to the study period. The meta-analysis of findings between the current two cohorts may also have masked some specific findings of note: for instance, HPV16 and EBV had the strongest associations with mortality risk in UKB, but neither association was present in NSHD. If these were not chance findings in UKB, it may be that these pathogens are associated with mortality at younger ages (middle age) as opposed to older age as studied in NSHD.

Total pathogen burden was associated with increased risk of frailty and all-cause mortality in minimally adjusted models, Multiple previous studies have found a positive association between pathogen burden and mortality^16^^,^^45^ and frailty-related biomarkers.^17^ However, in these studies, all pathogens were given equal weighting regardless of pathogenicity, which risks underestimating the impact of the most detrimental pathogens. This would have been especially true for the current study due to the greater number of pathogens studied had we not created inflammation-weighted PBIs to counteract this concern. This led to significantly stronger relationships with frailty compared to the unweighted counterpart model, validating the importance of our novel approach and potentially reflecting the relative importance of inflammation in ageing.

Our study results are bolstered by several strengths. It is the largest to date in terms of number of pathogens examined (17 agents) and included a large, combined sample of 11 398 total participants. This allowed us to detect novel relationships between seropositivity and frailty. We used data from two large and well‐characterized population‐based cohorts which allowed the creation of a detailed FI encompassing a wide range of physiological domains with similar items across the two cohorts. Such indices have been associated with a number of relevant outcomes such as mortality and hospitalization.^4^^,^^5^ The use of multiplex serology data to directly ascertain pathogen exposure avoids the potential inaccuracies associated with either self-report of past infection or relying on diagnosis of potentially asymptomatic infections from linked electronic health records. Finally, the use of linked mortality records ensured accurate mortality data over a long follow-up period.

However, several limitations require discussion. First, while the cohorts are community samples, they are not representative of the UK population. Participants remaining in NSHD were only broadly representative of the study population at recruitment and selective attrition will have further biased the sample.^20^ Participants in UKB are healthier and more socially advantaged than the UK population,^46^ however associations between risk factors and mortality in this cohort are similar to more representative samples.^47^ Secondly, our analyses are observational and, in the case of the frailty analyses, cross-sectional. Associations may arise from reverse causality or residual/unmeasured confounding, particularly for the smaller number of infections which are more commonly obtained in adolescence and adulthood. There is also evidence that frailty may increase the risk of recent infection or loss of seropositivity.^48^^,^^49^ Third, despite our large sample size, some findings lacked precision where seropositivity for pathogens was either very common or very infrequent. Alongside the somewhat limited number of mortality events, we chose not to examine associations of seroreactivity levels for antigens with each outcome, for which testing may have suffered from limited statistical power. Fourth, serostatus for this set of pathogens was only assessed once and individuals may have seroconverted in either direction either before or after serological assessment. We were unable to retrospectively identify the age or severity of infection, limiting investigation of these factors in relation to the outcomes. Clearly, we also cannot rule out the potential impact of other non-measured pathogens. We also note that the COVID-19 pandemic emerged some years after baseline samples were taken and that COVID-19 will have been an important subsequent cause of death and have been included in total mortality counts.

In conclusion, our findings add to evidence suggesting that exposure to multiple pathogens may be detrimental to lifelong health, even in the absence of symptomatic disease. We find that *H. pylori* and *T. gondii* may warrant targeting beyond their immediate acute infections and other pathogens such as *C. trachomatis*, HSV1, EBV, and CMV may also merit further investigation. Given this is the first investigation of many of these pathogens, our findings require confirmation in larger datasets. Such studies could also establish or refute some of the more marginal associations we observed and enable the testing of the relevance of seroreactivity (recent activity against pathogens) for these outcomes. Given that seropositivity for several pathogens appears to be a risk factor for frailty and/or mortality risk, future research should also focus on assessing whether or not these associations represent causation, for example through other study designs that aid causal inference, such as Mendelian randomization.^50^ This would help to motivate whether preventative measures or treatments for relevant pathogens could be tested as interventions to promote healthy ageing.

## Supplementary Material

glag046_Supplementary_Data
